# Microstructure, Transport, and Mechanics of Compacted Clay Simulated at the 0.1 μm Scale (1400 Smectite Clay Particles) Using a Coarse-Grained Model with Explicit Counterions

**DOI:** 10.1021/acs.jpcc.6c00004

**Published:** 2026-03-03

**Authors:** Xiaojin Zheng, Ian C. Bourg

**Affiliations:** † Department of Civil and Environmental Engineering, Princeton University, Princeton, New Jersey 08544, United States; ‡ High Meadows Environmental Institute, 6740 Princeton University, Princeton, New Jersey 08544, United States

## Abstract

Clay-rich geomaterials play a critical role in many subsurface systems. The macroscale properties of these materials (low permeability, high ionic conductivity, high swelling pressure, etc.) are sensitive to molecular-level adsorption and hydration interactions at clay–water interfaces. Efforts to develop multiscale simulation approaches to predict these properties reveal a scale gap between atomistic simulations (typically limited to systems smaller than 10 nm) and continuum-scale models (which use computational grid elements with dimensions ≳ 10 μm). In this study, we present a coarse-grained (CG) framework that partly bridges this gap by simulating compacted smectite clay assemblages with dimensions of 0.1 μm containing 1,400 clay particles across a range of dry densities (1,050 to 1,850 kg·m^–3^) and Na/Ca counterion compositions (Na fraction ranging from 0.2 to 1). The simulated systems, along with their reconstructed binary three-dimensional pore networks, are used to evaluate the microstructure, pore size distribution, tortuosity, ion diffusivity, and swelling pressure of compacted smectite clay. Results show that our approach captures important features of the mesoscale heterogeneity of compacted clays, including tactoid formation, hierarchical porosity, and anisotropic pore networks. Results also reveal how compaction and counterion composition govern emergent behaviors, including dominant pore sizes, directional transport, and electrochemical response. This work highlights the potential of CG simulations to bridge molecular and continuum scales and to advance geotechnical and environmental applications involving clay-rich materials as well as related nanoporous media such as geopolymers and calcium-silicate-hydrate. However, the results also suggest that accurate prediction of certain microstructural and mechanical properties (e.g., swelling pressure) may require even larger-scale systems on the order of 1 μm.

## Graphical abstract

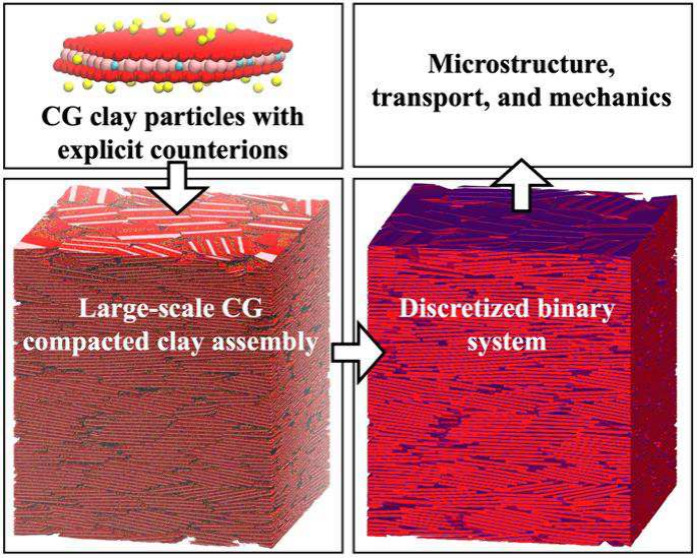

## 1 Introduction

Clay minerals—phyllosilicate minerals such as smectite, Illite, and kaolinite—are fundamental components of soils, sediments, and engineered barriers with important impacts in environmental sustainability and energy security.^1,2^ Their unique properties, including low permeability, high swelling pressure, high ionic conductivity, and ductile mechanics, underpin applications ranging from nuclear waste containment and geologic carbon storage to contaminant remediation and subsurface hydrology.^3–6^

Despite the importance of clay minerals outlined above, our understanding of clay systems remains limited by significant scale gaps. A core challenge lies in relating their behaviors observed at micrometer to meter scales—such as aggregation, pore connectivity, and hydraulic properties—to fundamental interactions occurring at the nanometer scale, including electrostatic forces, van der Waals interactions, and ion-specific effects.^7,8^ Experimental techniques such as small-angle X-ray scattering (SAXS) and mercury intrusion porosimetry offer valuable nanoscale insights but are constrained by limited spatial resolution, insensitivity to anisotropy, and difficulty in decoupling overlapping physical mechanisms.^9–11^ In contrast, molecular dynamics (MD) simulations provide atomistic details on processes such as ion adsorption, surface hydration, interparticle interactions, and transport properties.^12–15^ However, conventional all-atom MD simulations are computationally prohibitive and typically restricted to systems containing a few tens of clay particles.^16–18^ This scale limitation prevents them from predicting emergent mesoscale phenomena like the formation of tactoids (i.e., stacks of parallel clay particles) and macropores.^19–23^ These well-known limitations of experimental and computational molecular scale approaches hinder the development of predictive models for clay-rich materials.

Advances in coarse-grained (CG) modeling offer a path to bridge the scale gap outlined above.^24^ By accelerating sampling of particle-scale dynamics and reducing computational cost, CG simulations can accommodate systems with hundreds to thousands of clay particles.^25–27^ However, most CG models developed for clay systems fail to capture essential features of clay interparticle interactions, such as the coexistence of discrete crystalline and osmotic swelling states at certain dry densities or salinities.^1,28,29^ Furthermore, models that rely on implicit ions are unable to represent feedbacks between electrical double layer (EDL) structure and clay aggregation and are inherently valid only for a single salinity and counterion type.^30,31^ A recently developed clay CG model^32,33^ parametrized to match all-atom MD simulations overcomes these limitations by explicitly reproducing short-range interparticle forces and EDL structures with Na^+^ and/or Ca^2+^ exchangeable cations. The model enables robust analysis of clay aggregation and tactoid formation.^32,33^ However, previous studies using this model focused on relatively low solid volume fractions (up to 6%), corresponding to loosely structured clay gels.

In this study, we employ the new CG model described above to investigate how increasing dry density influences the microstructure, transport, and mechanical properties of systems containing thousands of particles. Specifically, we simulate montmorillonite assemblages comprising up to 1,397 clay particles (25 nm in diameter) with explicit representation of mixed Na^+^/Ca^2+^ counterions across a wide range of dry densities. This approach establishes the link between nanoscale interactions and emergent mesoscale heterogeneity, providing detailed predictions of clay microstructure, pore size distribution, tortuosity, and swelling pressure across density regimes relevant to engineered barriers and clay-rich sediments and sedimentary rocks.

We focus on addressing two key questions related to multiscale simulations of colloidal clay systems. First, what length scale is required in atomistic or particle scale simulations to capture experimentally observed continuum-scale properties of colloidal assemblages? Specifically, can simulations of thousands of clay particles reproduce continuum-scale properties of smectite, or are even larger systems required to resolve important features?^34^ Second, what particle-scale information is necessary to predict the Darcy-scale properties of smectite clay?^35^ In particular, can microstructural, transport, and mechanical properties be inferred solely from colloidal particle positions, or does successful upscaling require explicit consideration of counterion distribution and dynamics in EDLs surrounding charged clay particles? Addressing these questions is crucial to enable multiscale modeling of colloidal systems in complex environments.^36,37^

## 2 Methodology

### 2.1 System Buildup

#### 2.1.1 Coarse-Grained Molecular Dynamics Simulations

Smectite clay is composed of flake-shaped lamellae with a thickness of 0.94 nm and diameters of ∼25 to 250 nm.^38^ In this study, we simulate montmorillonite—the prototypical smectite mineral—using a representative unit cell formula: $\left(Ca_{0.4-0.4f_{\mathrm{Na}}}Na_{0.8f_{\mathrm{Na}}}\right){\mathrm{Al}}_{3.2}{\mathrm{Mg}}_{0.8}{\mathrm{Si}}_{8}O_{20}{\left(\mathrm{OH}\right)}_{4}$ . Here, *f*_Na_ is defined as the fractional contribution of Na ions to the total exchangeable cation charge: *f*_Na_ = *z*_Na_ *n*_Na_/(*z*_Na_ *n*_Na_ + *z*_Ca_ *n*_Ca_), where *z*_*i*_ and *n*_*i*_ are the valence and mole number of counterion *i*, respectively. The simulated clay particles carry a negative structural charge of 0.8 e per unit cell (equivalent to 105 C·g^–1^), resulting from randomly distributed isomorphic substitutions of octahedral Al by Mg. This negative charge is exactly balanced by Ca and Na counterions, i.e., the simulated systems contain no excess salt. Because of the random distribution of isomorphic substitutions, natural smectites exhibit charge heterogeneity both within and between particles. For simplicity, we neglect the second effect, i.e., every simulated particle carries the same heterogeneous charge distribution. We expect that this simplification has only a minor impact on microstructural, transport, and mechanical properties of compacted clay. Isomorphic substitutions of tetrahedral Si by Al, the predominated type of structural charge site in illite and certain smectites (e.g., beidellite), would localize charge closer to the basal surfaces, possibly impacting certain properties, such as swelling mechanics and ion diffusion. Their impact was not evaluated in this work, as the CG model was parametrized using MD simulations of a typical montmorillonite with only octahedral substitutions. Future work will aim to derive CG interaction potentials for hydrated clay minerals with tetrahedral charge sites.

Instead of modeling each atom explicitly as in all-atom MD simulations, we employ a newly developed CG model introduced by Shen et al. (2025)^32^ and Zheng et al. (2025).^33^ This model incorporates two key simplifications: first, it represents only half of the clay atoms (specifically, structural Al and Mg atoms, surface O atoms, and Na and Ca exchangeable cations); second, it treats water implicitly as a uniform dielectric continuum. These simplifications significantly reduce computational cost and enable simulations at larger spatial and temporal scales, while the explicit representation of exchangeable cations enables the model to capture EDL structures. The CG effective interaction potential model uses a screened Coulomb interaction supplemented by tabular potentials parametrized to match all-atom MD simulations of ions and smectite particles in liquid water carried out using the CLAYFF model of clay minerals,^39^ the SPC/E water model,^40^ and the Dang-Smith and Åqvist models for Na and Ca.^41,42^ These all-atom interatomic potential models have been extensively validated against experimental data on hydrated phyllosilicate properties.^17,43,44^ As a result, the CG model is expected to accurately capture short-range interparticle interactions and EDL structures, with fidelity approaching that of all-atom MD simulations. A detailed description of the CG force field development is available in Shen et al. (2025)^32^ and Zheng et al. (2025).^33^

Using this new CG model, we generate systems containing thousands of clay particles with explicit Ca and Na counterions (*f*_Na_ = 0.2). The initial simulation cell measures 100 × 100 × 200 nm. To populate this system, hexagonal clay particles with a diameter of 25 nm are inserted into the simulation cell at randomly selected positions with a parallel orientation using the *insert-molecules* command of the GROMACS MD simulation program. Although different initial configurations may lead to variations in local microstructure, their impact is limited because staged equilibration and progressive uniaxial compaction allow platelet rearrangement and tactoid formation to emerge naturally. Previous all-atom MD simulations of analogous (though necessarily smaller) systems showed that the use of replica-exchange MD (REMD) simulations to accelerate configurational sampling had minimal impact of the resulting platelet configurations.^44^ The size of the simulated particles in directions parallel to the clay basal surface is ∼ 10 times smaller than that of natural smectite particles,^45^ but matches that of a synthetic smectite used as a reference colloidal material.^46^ Our previous CG simulations have shown that 25 nm-diameter particles are sufficiently large to mimic the properties of smectite particles typically found in bentonite.^33^ The maximum number of particles that can be inserted into the simulation cell is 1,397, corresponding to an initial dry density of 1031 kg·m^–3^, a solid volume fraction of 0.36, and a total of approximately 23 million interaction sites. For comparison, the equivalent all-atom MD simulation would contain about 185 million atoms.

The system is then progressively compacted along the *z*-direction at 298 K using the LAMMPS MD simulation program by gradually decreasing the simulation cell size. Because water is modeled implicitly as a dielectric continuum, the pore space is inherently fully saturated at all times. Compaction therefore occurs under fully saturated, drained conditions. During compaction, changes in water content reflect variations in pore volume. Conversely, the abundance of ions remains constant during compaction as these are explicitly represented. This constant abundance of ions corresponds to the expected behavior for systems that contain no excess salt. The compaction continues until a final dry density of approximately 1850 kg·m^–3^ is reached. Five dry density states along this deformation path—1031, 1215, 1416, 1640, and 1843 kg·m^–3^—are selected for further equilibration followed by detailed analysis.

To investigate the impact of counterion composition on clay aggregation, we use the same methodology to construct systems with *f*_Na_ = 0.6 or 1.0 containing 1,367 or 1,366 particles, respectively. The system size, particle size, and dry densities selected for further equilibration and analysis are identical to those used in the *f*_Na_ = 0.2 case. In total, we analyze 15 distinct cases representing different combinations of dry density and counterion composition (*f*_Na_ = 0.2, 0.6, and 1). Figure 1 shows representative configurations for *f*_Na_ = 0.2 equilibrated at dry densities of 1030, 1416, and 1843 kg·m^–3^. Figure 2 shows representative configurations for *f*_Na_ = 0.2, 0.6, or 1.0 at ρ_d_ = 1620 kg·m^–3^.

**Figure 1:**
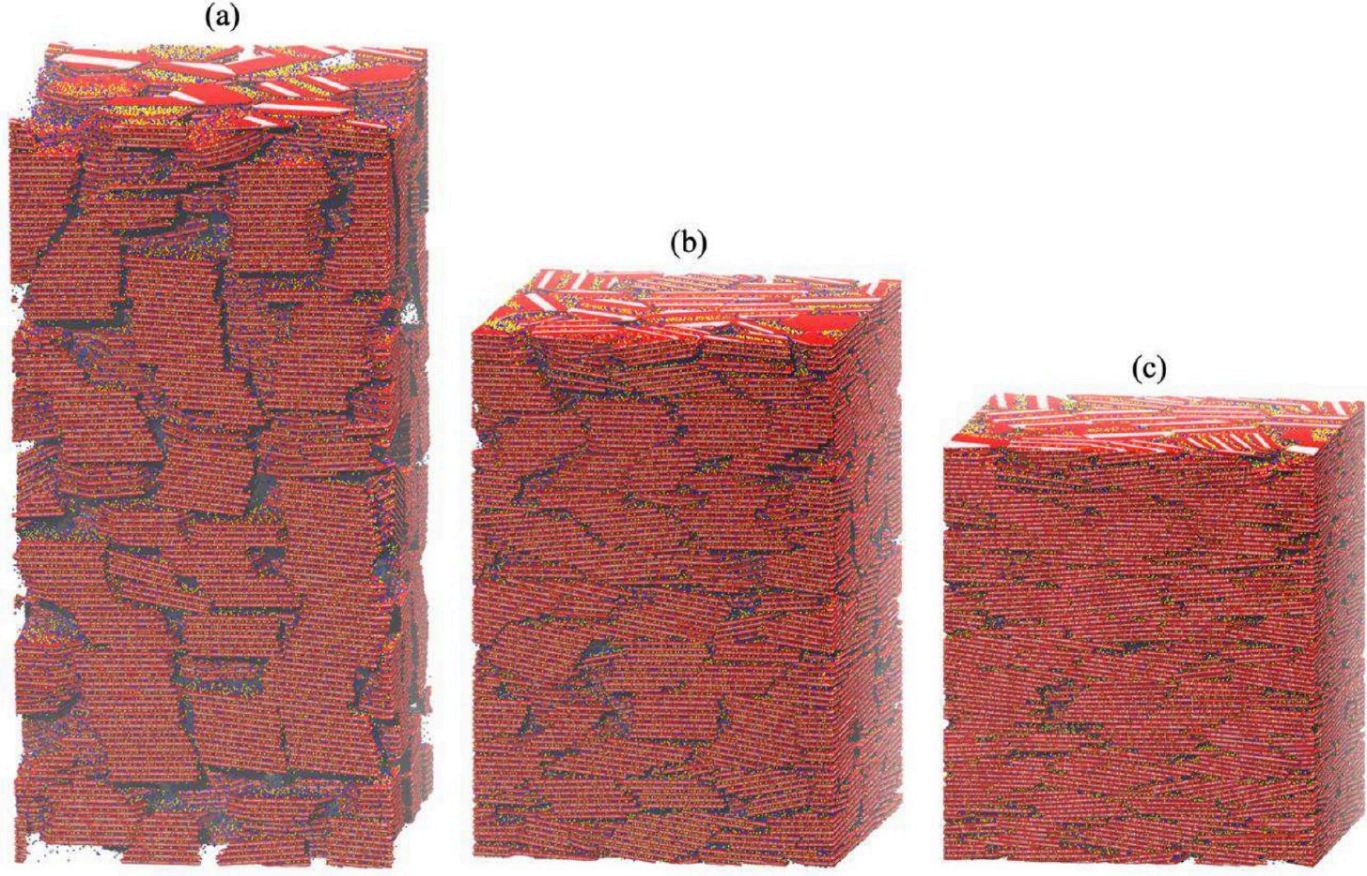
Snapshots of large-scale smectite assemblages with Na and Ca exchangeable cations (*f*_Na_ = 0.2) at varying dry densities (ρ_d_): (a) ρ_d_ = 1030 kg·m^–3^; (b) ρ_d_ = 1416 kg·m^–3^; (c) ρ_d_ = 1843 kg·m^–3^. Each system contains 1397 hexagonal clay particles with a diameter of 25 nm. The system in Figure 1a has dimensions of 100 × 100 × 200 nm^3^. Empty space represents implicitly modeled water. Clay Mg, O, and Al atoms are shown as pink, red, and cyan spheres, respectively; exchangeable Ca and Na ions are shown as yellow and blue spheres, respectively.

**Figure 2:**
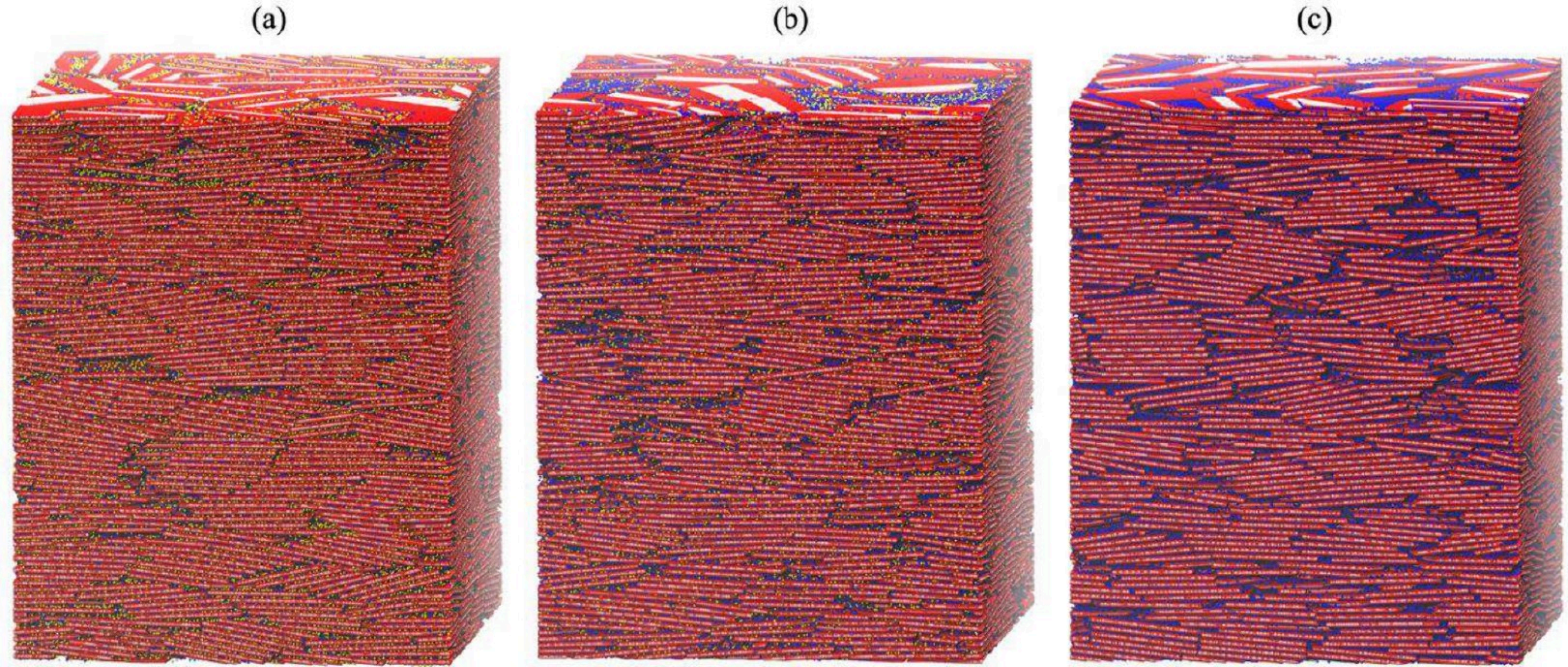
Snapshots of large-scale smectite assemblages with Na and/or Ca exchangeable cations (ρ_d_ = 1620 kg·m^–3^) at various Na fractions (*f*_Na_): (a) *f*_Na_ = 0.2; (b) *f*_Na_ = 0.6; (c) *f*_Na_ = 1.0. Empty space represents implicitly modeled water. Colors have the same meaning as in Figure 1.

In all cases, water is modeled implicitly by applying a uniform static dielectric constant calculated based on the explicit SPC/E water model.^47^ Hydration forces and ion-specific effects are incorporated through effective ion–surface and ion–ion interparticle potentials parametrized against all-atom MD simulations in explicit water, while explicit hydrogen-bond dynamics and discrete water rearrangements are not resolved in the implicit solvent representation. Explicit ions are essential for capturing the dynamic redistribution of Na^+^ and Ca^2+^ under evolving confinement during compaction, which in turn governs pore geometry and fabric. For a 25 nm platelet, the numbers of exchangeable ions per clay particle are 161 Na^+^/322 Ca^2+^ (*f*_Na_ = 0.2), 483 Na^+^/161 Ca^2+^ (*f*_Na_ = 0.6), and 805 Na^+^/0 Ca^2+^ (*f*_Na_ = 1.0). For the systems simulated here, the total number of counterions is on the order of 10^6^. This causes a significant increase in computational cost (by a factor >2) compared to simulations with implicit ions, particularly because of the cost associated with the reciprocal-space calculation of long-range Coulomb interactions. While an implicit-ion potential of mean force (PMF) would be more computationally efficient and would reproduce averaged Na/Ca interaction effects, it would not capture dynamic ion redistribution effects or their coupling to the evolving pore geometry during compaction. These include, for example, the tendency of Ca ions in mixed Na/Ca-smectite systems to preferentially adsorb on internal vs external tactoid surfaces, as observed experimentally^48^ and in our previous CG simulations of smectite clay suspensions.^33^ Future side-by-side comparison of implicit and explicit ion treatments in mixed-cation systems represents an interesting opportunity for future work. The masses of Na^+^ and Ca^2+^ are set to their physical values (22.990 and 40.078 Da, respectively). The Al, Mg, and O interaction sites represent coarse-grained pseudoatoms rather than individual atoms; their assigned molar masses (53.152, 50.476, and 42.170 Da, respectively) are chosen to ensure that the total mass of each clay platelet matches that of the corresponding montmorillonite particle, thereby preserving the prescribed dry density.

The equations of motion for ions and clay atoms are integrated using a Langevin thermostat, which applies random forces and frictional drag at each time step to mimic solvent effects.^49^ To accelerate equilibration, the damping factor of the thermostat is increased by a factor of 10^5^ for both the ions and clay particles, an approach that essentially lowers the viscosity of the implicit aqueous solvent. Coarse-grained simulations are performed using the LAMMPS code with a time step that is increased from 0.1 to 15 fs as equilibration progresses. A short-range cutoff of 50 Å is used, and long-range Coulomb interactions beyond this cutoff are treated using the particle–particle particle-mesh (PPPM) method. Equilibration simulations are conducted in the NVE ensemble at a temperature of 298 K. For each case, the equilibration process takes approximately 1.5 to 2.6 ns, depending on the system’s dry density and counterion composition. In total, the simulations for all 15 cases required approximately 3.2 million CPU hours.

We note that the systems simulated in this study are idealized in a few important ways. First, the clay particles are treated as rigid for simplicity, following Zheng et al. (2025).^33^ In our previous simulations of dilute clay systems that used flexible particles, only minimal bending was observed.^32^ In the more densely packed systems examined here, the assumption of particle rigidity represents a more significant approximation that deserves further investigation in future work.^50^ Second, the simulated clay particle size is uniformly set to 25 nm, comparable to that of synthetic laponite but smaller than natural smectite particles, which typically exhibit diameters approximately an order of magnitude larger. The smaller platelets simulated here are likely to exhibit enhanced stacking due to their larger edge-specific surface area and reduced long-range face-to-face repulsive interactions.^33^ Moreover, the natural polydispersity of clay particle sizes in real smectite, which may further reduce pore sizes, is not considered in the present study and should be addressed in future work. Third, compared with conventional all-atom molecular dynamics simulations, the total number of platelets considered here (up to 1,397) exceeds the characteristic length scales of tactoids,^51^ thereby enabling the emergence of mesoscale features such as hierarchical porosity in compacted smectite. However, natural smectite assemblages can exhibit broad particle-size distributions and structural heterogeneity extending to micrometer scales; future work will examine the associated finite-size effects. Finally, dehydration in this model is initiated by inserting parallel particles, followed by relatively rapid forced compaction until a high dry density is achieved. This procedure may influence the degree of stacking. The pronounced effects of Na versus Ca on tactoid size observed in dilute smectite suspensions^33^ are less evident in the compacted systems studied here, where configurational rearrangements are constrained by limited space.

#### 2.1.2 System Discretization

To facilitate the evaluation of pore structure in the simulated systems, each equilibrated configuration obtained in MD simulations is discretized onto a three-dimensional (3D) grid. The grid spacing (Δ*d*) is set to 2 Å, resulting in a 3D array with dimensions of up to 500 × 500 × 1000 for the initial (predeformation) systems. This pore space array, referred to hereafter as *P*_*s*_, is a binary matrix where each element is assigned a value of 1 to represent pore space or 0 to represent clay. To generate the binary representation for systems containing thousands of clay particles—each modeled as a hexagonal prism—a multistep workflow is employed. For each particle, the code first uses its atomic coordinates to determine the centroid, the vector normal to the basal surface, and the in-plane orientation angle. Next, the code evaluates whether each grid point lies within the prism. This involves an axial check to determine if a point falls within the height of the prism along the central axis, and a radial check to determine whether the point lies within the hexagonal cross-section on the basal plane. Special attention is paid to particles that intersect the periodic boundaries to ensure continuity across system edges. Once the *P*_*s*_ binary array is constructed, it serves as the basis for reconstructing the 3D pore structure of each system. Figure 3 presents reconstructions of three representative systems based on this method.

**Figure 3:**
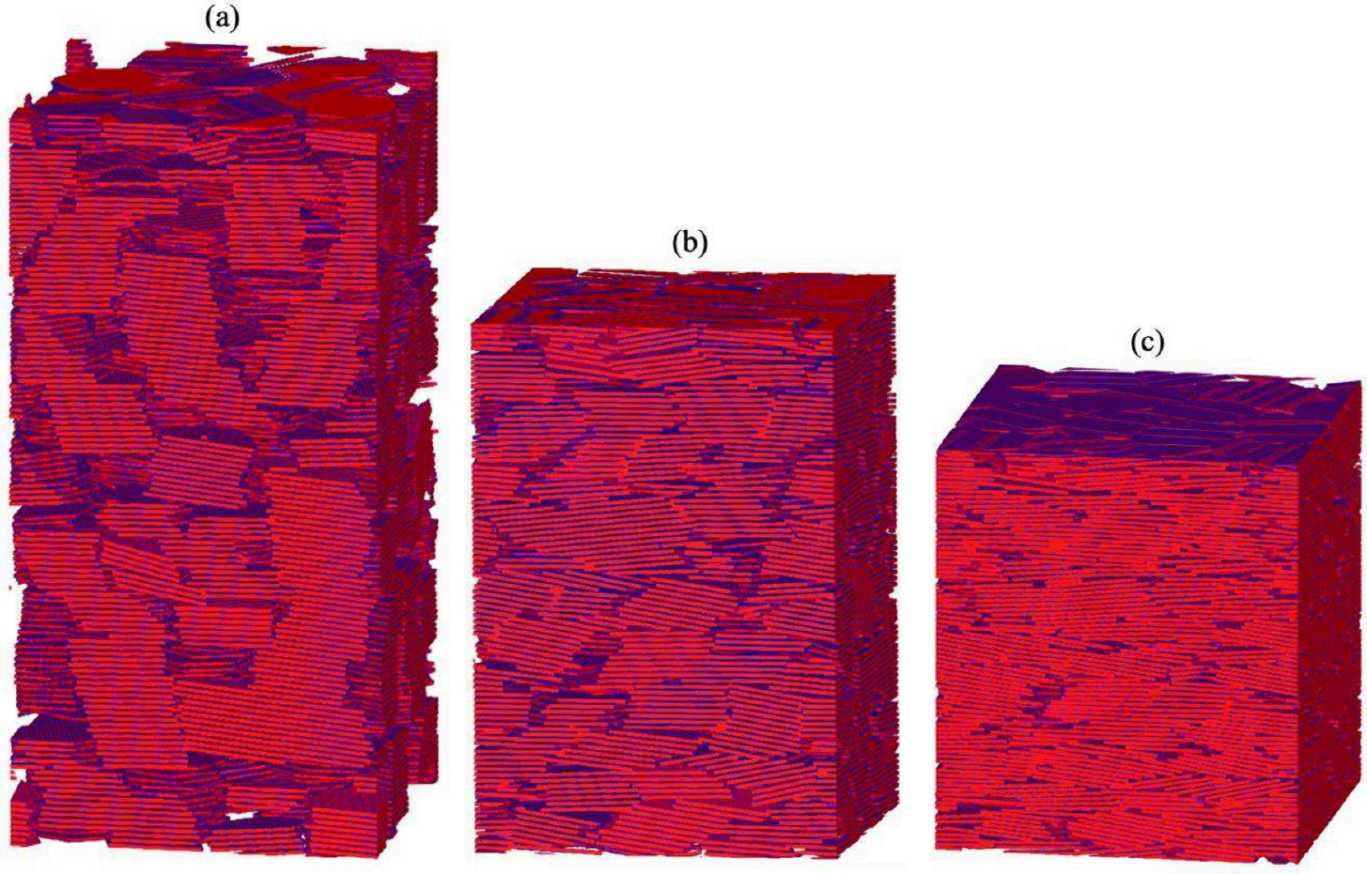
Reconstructed smectite assemblages corresponding to the configurations shown in Figure 1: (a) ρ_d_ = 1030 kg·m^–3^; (b) ρ_d_ = 1416 kg·m^–3^; (c) ρ_d_ = 1843 kg·m^–3^. Each system is reconstructed by discretizing the equilibrated MD configuration into a three-dimensional binary array, where 1 represents pore space and 0 represents clay.

### 2.2 Property Computation

#### 2.2.1 Clay Microstructure

Small-angle X-ray scattering (SAXS) experiments are widely used to investigate the microstructure of clay minerals and other layered colloidal materials by analyzing the relationship between scattering intensity (*I*) and the scattering vector magnitude (*q*).^52–57^ In this study, we offer an alternative approach to generate the *I*–*q* relationship using the reconstructed clay systems represented by the 3D binary array (*P*_*s*_). This computational method provides valuable microstructural information comparable to experimental SAXS data.^58,59^ To extract the *I*–*q* relationship from the reconstructed systems, we first compute the three-dimensional Fourier Transform (*FT*) of the binary array. This transform captures the spatial frequency representation of the pore structure and is defined as

1 $$FT=\overset{N_{x}-1}{\underset{x=0}{\sum }}\overset{N_{y}-1}{\underset{y=0}{\sum }}\overset{N_{z}-1}{\underset{z=0}{\sum }}P_{s}\left(x,y,z\right)e^{-i\left(k_{x}x+k_{y}y+k_{z}z\right)}$$

where *k*_*x*_, *k*_*Y*_, *k*_*z*_ are the frequency components in the x, y, and z directions, and *N*_*x*_, *N*_*Y*_, *N*_*z*_ are the number of grid points along these directions. The scattering intensity is then calculated as the squared magnitude of the Fourier Transform (*I* = |*FT*|^2^).

Next, the scattering vector components in reciprocal space are computed using the dimensions of the simulation box and the Fourier indices: $q_{x}=\frac{2\pi }{L_{x}}k_{x}$ , $q_{y}=\frac{2\pi }{L_{y}}k_{y}$ , $q_{z}=\frac{2\pi }{L_{z}}k_{z}$ , where *L*_*x*_, *L*_*y*_, *L*_*z*_ are the box lengths in each direction, and *k*_*x*_, *k*_*y*_, *k*_*z*_ are integer indices ranging from −*N*/2 to *N*/2, where *N* is the number of grid points along that dimension in the *P*_*s*_ array. The total magnitude of the scattering vector is then given by $q=\sqrt{q_{x}^{2}+q_{y}^{2}+q_{z}^{2}}$ .

To construct the *I*–*q* curve, the computed intensity values are grouped into bins based on ranges of *q*, and the intensities within each bin are summed via *I*_*bin*__,*i*_ = ∑_*q*_*i*_ ≤ *q* ≤ *q*_*i*__+1__ *I*(*q*). The resulting *I*–*q* curve typically exhibits distinct peaks that correspond to structural features of clay platelets.^60^ The basal spacing (*d*_001_) is extracted from the position of the primary peak via *d*_001_ = 2π/*q*_peak_, where *q*_peak_ is the location of the maximum intensity. Another key feature is the half-width at half-maximum (*w*) of the scattering peak, which relates to the number of clay platelets per tactoid as *N*_platelet_ ≅ *q*_peak_/*w*.^52,59^ In the low-*q* region, the scattering intensity often follows a power-law decay of the form *I*(*q*) ∝ *q* ^–*x*^, where the exponent *x* provides structural insights into the degree of aggregation or ordering within the sample.^22,61^

#### 2.2.2 Pore Size Information

The chord length distribution method offers a simple approach to characterize pore size distributions from binary voxel data.^19,62–64^ From the *P*_*s*_ array for each reconstructed system, the porosity (ϕ) is directly calculated by counting all grid elements with a value of 1 (representing pore space) and dividing by the total number of grid elements. To obtain the pore size distribution, a bisecting vector is defined along a chosen direction, where each element is either 1 (pore) or 0 (solid matrix). This vector is segmented into continuous subvectors, or chords, representing uninterrupted lengths of pore space. The lengths of these chords are then compiled into a data set, forming a directional pore size distribution. The bisection is uniformly applied along the x-, y-, and *z*-directions, with the total number of bisecting vectors equal to *N*_*x*_ *N*_*y*_ + *N*_*x*_ *N*_*z*_ + *N*_*y*_ *N*_*z*_, where *N*_*x*_, *N*_*y*_, *N*_*z*_ are the numbers of grid points along each axis. By systematically sweeping along these predefined directions and recording uninterrupted pore segments, the method captures key directional statistics of the pore structure without requiring complex geometric analysis. Its advantages include computational efficiency, ease of implementation, and scalability for analyzing large 3D data sets.

Experimentally, mercury intrusion capillary pressure (MICP) testing is widely used to characterize the pore connectivity and size distribution of porous materials.^65,66^ The experimental approach is typically limited to pores larger than 2 to 6 nm^67^ because of the high pressures required to displace water from smaller pores. Here, we apply a computational approach that mimics the mercury invasion phenomenon, without the length scale limitation. Specifically, our approach consists in continuously inserting cubic boxes—representing mercury—into the reconstructed pore structure from the lower boundary of the system. The detailed workflow is illustrated in Figure S3 of the Supporting Information, which shows how the invaded region propagates stepwise during the simulation. In this example workflow, the insertion box is a cube with a side length of 3 cells (i.e., a cube with 3 × 3 × 3 cells), and the program randomly selects an initial invaded region of that size at the bottom plane of the simulated system. Then, the algorithm examines all boundary facets of this initial invaded volume—totaling 3 × 3 × 6 = 54 facets—to determine whether any are in contact with a neighboring pore of the same size. If a facet is in direct contact with such a pore and is centrally aligned with one of its faces (as shown in Step 1 of Figure S3), the pore is marked as connected, and all of its constituent cells are labeled as invaded. If no other pore connections are found, a new iteration begins to examine whether any newly exposed boundary planes are connected to uninvaded pores. This process continues iteratively until either the invaded region reaches the top surface of the system or no further growth occurs. Once intrusion for a given insertion box size is completed, the box size is incrementally increased and the simulation restarts from the lower boundary, mimicking the effect of lower injection pressures at which mercury can only invade larger pores. By systematically varying the insertion box size, we emulate a range of intrusion pressures and probe the distribution of pore sizes accordingly. In this study, insertion box sizes range from 4 × 4 × 4 to 18 × 18 × 18 cells, corresponding to equivalent pore diameters of approximately 0.8 to 3.6 nm.

#### 2.2.3 Tortuosity

Tortuosity (τ), a measure of the convoluted nature of the transport pathways, is an important property in fine-grained geologic materials. It can be quantified by comparing the bulk diffusivity of a solute (*D*_0_)—representing its diffusion in free space—to its apparent diffusivity (*D*_a_) within the porous structure.^68^ It is commonly defined as

2 $$\tau^{2}=D_{0}/D_{a}$$

Here, *D*_a_ is not directly computed from the MD simulations because adequately sampling transport through long or narrow conduits at the scale of thousands of clay particles would require expensive simulation time. Instead, we use stochastic random walk (RW) simulations to estimate ion diffusivity within the reconstructed binary pore structure.^69^ In this study, 100 random walkers are initially placed at random positions within the central region of each simulated system. At each time step, a walker attempts to move to a neighboring voxel in a randomly selected direction (*x*, *y*, or *z*) to capture isotropic diffusion. The limited angular resolution imposed by the voxel grid introduces directional correlation bias, which can be alleviated by refining the grid resolution. If the target voxel lies within the pore space, the walker’s position is updated according to *r*(*t* + Δ*t*) = *r*(*t*) + Δ*r*, where Δ*r* is a random displacement equal to the voxel length. The physical time step Δ*t* is estimated using the Einstein relation: $\Delta t=\frac{{\left(\Delta d\right)}^{2}}{6D_{0}}$ , where Δ*d* is the grid spacing and *D*_0_ is the ion self-diffusivity in bulk liquid water. In systems with both Na and Ca, Δ*t* is calculated using the weighted-average *D*_0_-value of the two ions: *D*_0_ = *f*_Na_ *D*_Na_^bulk^ + (1 – *f*_Na_)*D*_Ca_^bulk^, where *D*_Na_^bulk^ and *D*_Ca_^bulk^ are the respective bulk diffusivities. If a walker attempts to move into a solid voxel (clay), it remains in place for that step. If all walkers are temporarily blocked, time does not advance until at least one walker successfully enters a pore voxel. Periodic boundary conditions are applied to allow continuous transport, while unwrapped coordinates are used to calculate the mean square displacement (MSD). The MSD obtained from each simulation is averaged over time using multiple time origins. An example of the MSD-time relationship at *f*_Na_ = 0.2 and ρ_d_ = 1000 kg m^–3^ is shown in Figure S5a of the Supporting Information. Finally, the apparent diffusivity of ions (*D*_a_) is calculated from the MSD as a function of observation time (*t*) using the well-known Einstein relation:^70^

3 $$D_{a}=\frac{1}{2d}\underset{t\to \infty }{\lim}\frac{\frac{1}{N}\overset{N}{\underset{i=1}{\sum }}{\left(r_{i}\left(t\right)-r_{i}\left(0\right)\right)}^{2}}{t}$$

where *N* is the number of ions, *r*_*i*_ is the position vector of ion *i*, and *d* is the dimensionality. Here, *D*_a_ is an average diffusivity in a three-dimensional space, as presented in Figure S5b in the Supporting Information. The RW model approach described above has been validated in previous studies,^71,72^ where diffusion emerges from the explicit pore geometry by allowing random walkers to move on a discrete lattice. Other approaches to RW simulations include continuum-scale frameworks that solve macroscopic advection–dispersion equations (ADEs) and preserve flux continuity across sharp boundaries without relying on grid-based geometry.^73,74^ The voxel-based approach used in this work provides a clear physical interpretation and is well suited for investigating transport governed by microstructural features, although it may require high spatial resolution to minimize grid-induced artifacts.

Alternatively, tortuosity can be evaluated using the resistor network method, a well-established approach that draws on the analogy between ion transport and electrical conduction. In this method, tortuosity is interpreted as the degree to which the material geometry impedes electric current flow. The pore space is modeled as an electrical circuit, where each pore voxel (nonzero grid element) is treated as a unit resistor. An electric potential difference (Δ*U*) is applied across opposite faces of the system. The electric potential field throughout the pore network is then calculated using Kirchhoff’s current law, which requires that the sum of all currents entering a node equals zero. An iterative solver updates the potential at each node until a steady-state solution (*U*_*eq*_) is reached. Once the steady state is established, the total current (*I*) through the system is computed, and the effective resistance (*R*_eff_) is determined via *R*_eff_ = *U*_*eq*_/*I*. This effective resistance reflects how the tortuous geometry of the pore network hinders transport. Tortuosity is then related to the effective resistance and porosity through the relation:^63^

4 $$\tau^{2}=R_{\mathrm{eff}}\phi /R_{\mathrm{bulk}}$$

where ϕ is porosity and *R*_bulk_ represents the resistance of a reference material with straight, uniform paths. This method offers a direct and intuitive assessment of how geometric complexity impacts transport through the medium. In both the random walk and resistor network approaches, tortuosity calculations are performed on the reconstructed pore geometry and do not explicitly incorporate ion distributions; ions influence these metrics only indirectly through their impact on the equilibrated microstructure.

## 3 Results and Discussion

### 3.1 Clay Microstructure

Figure 4 presents the relationship between scattering intensity (*I*) and scattering vector magnitude (*q*) predicted from the simulated clay assemblies, together with experimental measurements for Na-exchanged smectite at different dry densities.^75–77^ To enable comparison across data sets spanning different intensity scales, all curves were normalized at a reference value of *q*_ref_ = 0.2 Å^–1^, then vertically shifted to separate the groups of data sets for easier visualization of the characteristic peaks. The color of each line indicates the dry density of the Na-bentonite and the simulated clay assemblies with *f*_Na_ = 1.0. The unprocessed *I*–*q* relationships for simulated systems with different *f*_Na_ values are provided in Figure S1 of the Supporting Information.

**Figure 4:**
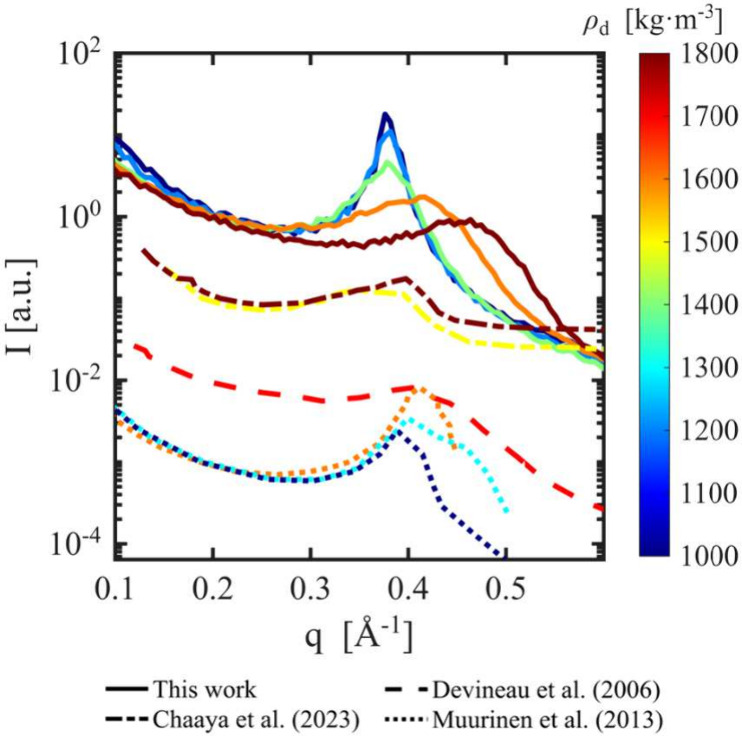
Scattering intensity (*I*) as a function of the scattering vector magnitude (*q*) for simulated clay systems with *f*_Na_ = 1.0 at different dry densities, along with experimental measurements on compacted Na-exchanged smectite.^75–77^ Different colors indicate different dry density conditions. Experimental data correspond to Kunipia-G smectite in a 10^–4^ mol/L NaCl solution compacted to dry densities of 1500 and 1800 kg·m^–3^ after 44 h;^75^ MX-80 smectite at 81% relative humidity compacted to 1700 kg·m^–3^;^76^ and MX-80 smectite compacted to 1000, 1300, and 1600 kg·m^–3^.^77^

The position and shape of the peaks in the *I*–*q* curves provide insight into the microstructure associated with the formation of clay tactoids. In general, narrower peaks correspond to more ordered structures with well-defined characteristic length scales, whereas broader peaks indicate more disordered configurations. As dry density increases, the particle arrangement becomes increasingly disordered (Figure 1). The presence of a single dominant peak in each curve suggests one predominant type of layer arrangement or pore size, consistent with previous SAXS observations.^52,58,59^ The ability to reproduce such *I*–*q* trends illustrates the capability of large-scale CG simulations to capture realistic clay microstructures (in particular, the existence of fluid-filled interlayer nanopores with a well-defined pore width) and to complement existing experimental approaches.

Following the methodology described in Section 2.2.1, the *I*–*q* curves shown in Figures 4 and S1 were used to compute the basal spacing (*d*_001_), as presented in Figure 5. The basal spacings captured by our CG model correspond to effective equilibrium hydration states rather than explicit representations of discrete hydration layers. The simulation results are broadly consistent with experimental measurements on core-scale compacted smectite samples. As expected, the basal spacing decreases with increasing dry density, reflecting the reduction of pore space. In general, *d*_001_ increases with increasing fraction of Na relative to Ca ions, since Na ions form a more extended diffuse ion cloud, whereas Ca ions predominantly adsorb in the immediate vicinity of the clay surfaces.^78^ However, the dependence of *d*_001_ on *f*_Na_ is less pronounced than in dilute clay systems,^33^ as also shown in Figure 2. This likely occurs because, under highly compacted conditions, the limited pore space restricts ion-mediated interactions that would otherwise affect clay stacking and aggregation. Consequently, while higher *f*_Na_ values correspond to larger *d*_001_ at ρ_d_ = 1000 and 1200 kg·m^–3^, the trend reverses at *ρ*_*d*_ = 1850 kg·m^–3^ (Figure 5).

**Figure 5:**
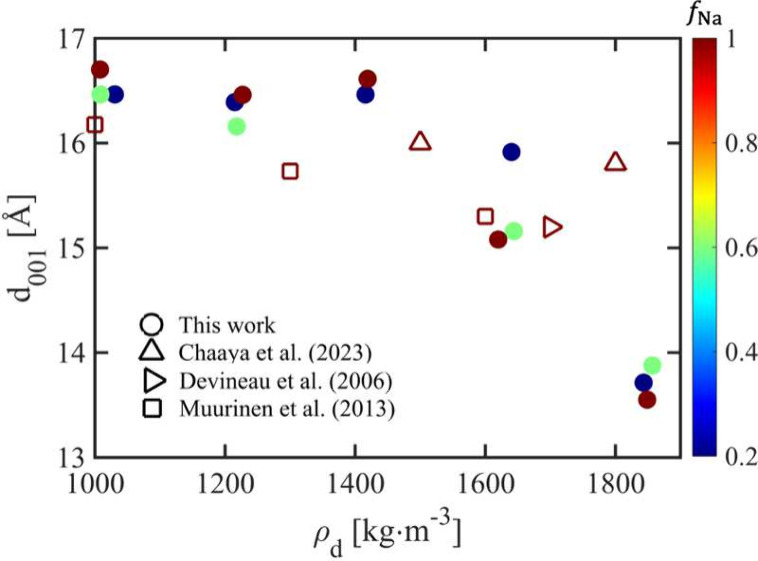
Basal spacing (*d*_001_) derived from the *I*–*q* relationships of the simulated clay assemblies and experimental compacted Na-smectite samples (Figure 4), shown as a function of dry density (ρ_d_) and Na fraction (*f*_Na_).^75–77^ The green marker at *ρ*_*d*_ = 1420 kg·m^–3^ is not visible due to overlap with the dark red marker.

Figure S2 in the Supporting Information summarizes other characteristic microstructural properties of the clay assemblies, including the number of platelets per tactoid (*N*_platelet_) and the exponent *x* of the power-law fit in the low-*q* region. As the system becomes more densely compacted, the stack size (i.e., the number of platelets per tactoid) decreases (Figure S2a) due to platelet sliding and increased stacking disorder.^33,79^ The exponent *x* is directly correlated with *N*_platelet_ and exhibits a similar decreasing trend from *x* ≈ 3.4 at *ρ*_*d*_ < 1300 kg·m^–3^ to *x* ≈ 2.1 at *ρ*_*d*_ < 1500 kg·m^–3^ (Figure S2b). In general, *x* provides insight into the mass fractal dimension: lower values (e.g., *x* ≈ 1) correspond to rod-like structures, intermediate values (*x* ≈ 2) to thin sheets, and higher values to densely aggregated morphologies.^80^ Our results are consistent with visual observations of the simulated structures (Figure 1) showing that the hierarchical structure of compacted smectite (with large tactoids separated by mesopores) is disrupted by compaction.

### 3.2 Pore Size Distribution

Pore size distribution is assessed using both quasi-mercury intrusion simulations and chord length analysis. Figure 6 presents the results of the quasi-mercury intrusion simulations, including snapshots of the invasion process in a simulated clay system (Figure 6a) and the mean invaded volume (*V*_invaded_) along with the differential volume contribution (dV_invaded_/d log­(D)) as a function of pore diameter (*D*) (Figure 6b). In these simulations, the side length of the insertion box is interpreted as the pore diameter. As expected, the invaded volume decreases with increasing dry density, indicating reduced pore connectivity and smaller average pore sizes. Red dots in Figure 6b indicate cases in which the invaded region reaches the top plane of the simulated system (i.e., fluid breakthrough) while blue dots represent cases where the invasion does not reach the top. The size of the largest insertion box associated with fluid breakthrough provides an estimate of the breakthrough pore size, which correlates with breakthrough pressure.^6^ We note that macroscopic clay samples may exhibit heterogeneity that exceed the scale of the microscale CG model, such that the breakthrough pore sizes determined here represent a lower boundary of the values exhibited by macroscopic clay samples, on the order of ∼15 nm for compacted bentonite clay.^81,82^

**Figure 6:**
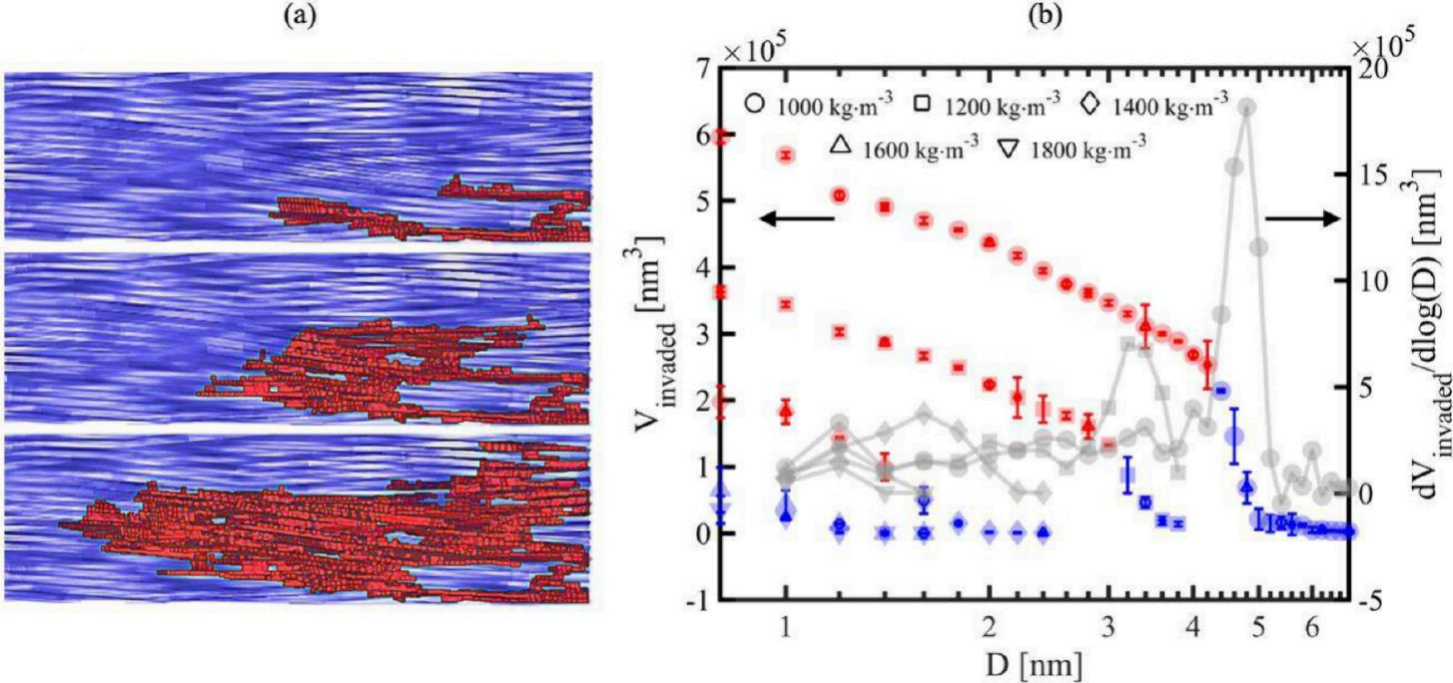
Results of quasi-mercury intrusion simulations. (a) Snapshots of the invasion process in a simulated clay system with ρ_d_ = 1850 kg·m^–3^; (b) mean invaded volume (*V*_invaded_) (colored markers) and differential volume contribution (dV_invaded_/d log­(D)) (gray lines) as a function of pore width (*D*) (i.e., insertion box size). For each microstructure, we performed 20 independent simulations by randomly selecting different initial invasion points at the lower boundary of the system. Each data point in the figure therefore represents the average of 20 simulations, with error bars indicating two standard errors. Red markers indicate cases where the invaded region reaches the top plane of the simulated system (i.e., successful breakthrough), while blue markers represent cases where the invasion does not reach the top. The five data sets in this figure correspond to five dry density conditions at *f*_Na_ = 0.2. Additional data sets for *f*_Na_ = 0.6 and 1.0 are provided in Figure S4 of the Supporting Information.

In our simulations, the total porosity is determined by subtracting the volume of clay particles from the system volume. The first point of each *V*_invaded_ line at *D* = 0.8 nm can be interpreted as the volume of intertactoid pores, given that the basal spacing does not exceed 1.7 nm (Figure 5). The percentage of intertactoid pores in the total pore volume (*f*_intertactoid_) calculated from these points equals 47% at ρ_d_ = 1000 kg·m^–3^ and decreases to 37%, 27%, 12%, and 9% as the density increases to 1850 kg·m^–3^. Table S1 in the Supporting Information reports the overall porosity (ϕ), intertactoid porosity (ϕ_intertactoid_), and intratactoid porosity (ϕ_intertactoid_) at different dry densities and Na fractions. The predicted ϕ_intertactoid_ values show excellent agreement with previous experimental measurements and CG simulation results. For example, at ρ_d_ = 1000 kg·m^–3^ and *f*_Na_ = 1.0, our prediction of ϕ_intertactoid_ = 0.33 closely matches the measured value of 0.32 for MX-80 smectite reported by Muurinen et al., 2013,^77^ as well as the CG prediction of 0.33 obtained using a probe radius of 0.75 nm by Zhang et al., 2023.^31^

Additionally, the slope of the curves in Figure 6b (gray lines), representing the differential volume contribution from pores of various diameters, provides insights into the distribution of pore sizes across the system. The pore diameter at the peak of each curve corresponds to the critical pore throat size. For instance, when ρ_d_ = 1000 kg·m^–3^ and *f*_Na_ = 0.2, the critical pore throat has a size of approximately 4.8 nm. As the dry density increases to ρ_d_ = 1800 kg·m^–3^, the peak shifts leftward and the critical pore throat size decreases to about 1.2 nm.

The chord length distribution method provides another perspective on pore size distribution. Figure 7 summarizes the relative frequency of pore diameters across six representative cases examined in this study. The red lines indicate the average pore size for each system. Note that chord length analysis is direction-dependent and in clay materials with strong anisotropy, chord length calculation along the x and y directions potentially yield unphysical results by overestimating the pore size. To address this, we report only chord lengths computed along the *z*-direction (i.e., the direction of compaction).

**Figure 7:**
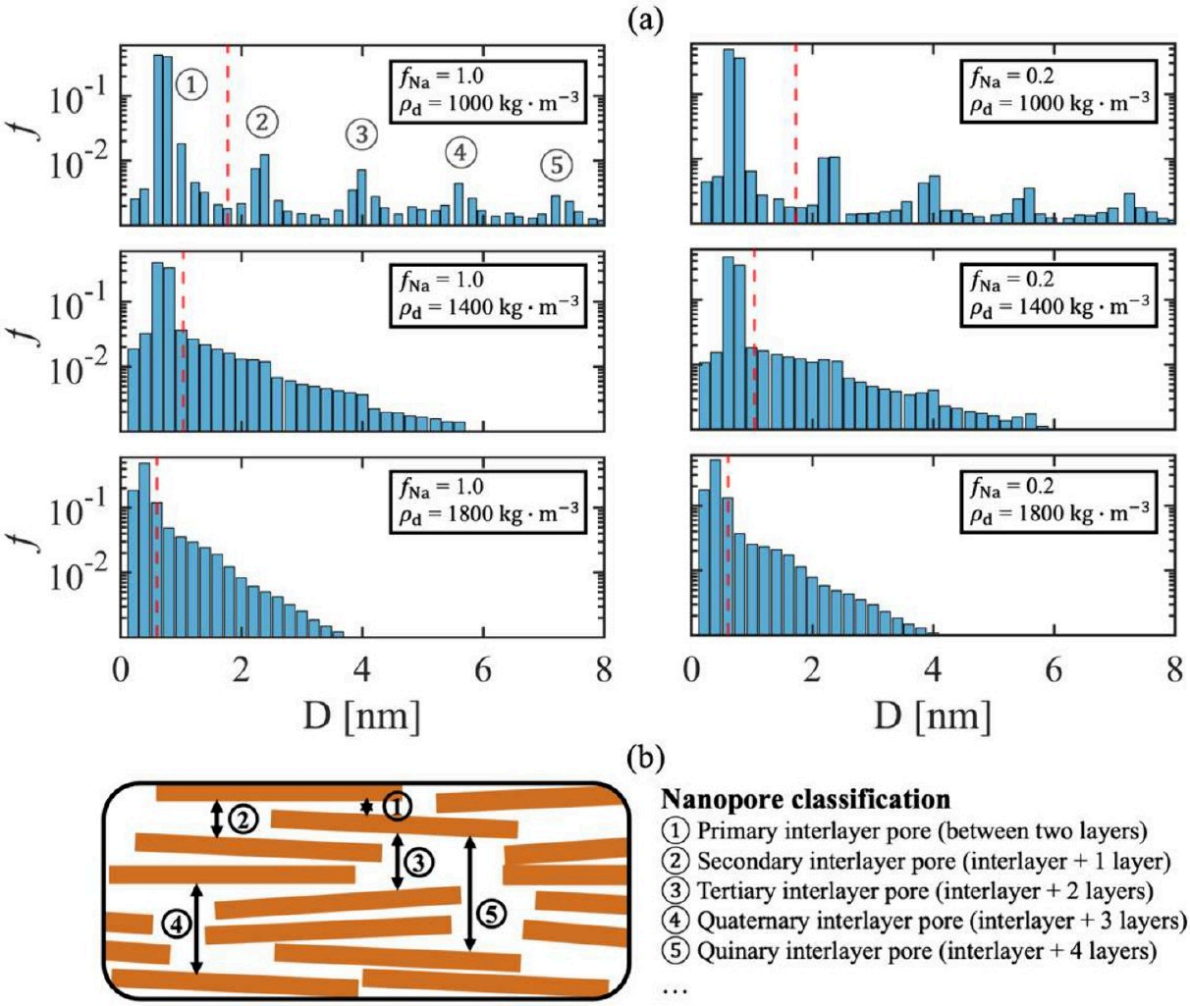
Pore size distribution in the direction normal to the clay particles determined by the chord length analysis method: (a) Relative frequency (*f*) of different pore widths (*D*) at different Na fractions (*f*_Na_) and dry densities (ρ_d_). Red lines indicate the mean pore size in each system. (b) Schematic illustration of the nanopore hierarchy in the simulated clay systems.

The pore size distribution in Figure 7 exhibit complex nanopore hierarchy. The first peak observed at *D* < 0.8 nm exhibits consistent high relative frequency, which physically represent abundant interlayer space between clay platelets within tactoids. The associated interlayer spacing is consistent with the basal spacing predictions in Figure 5, which shows that the inner spacing falls between 0.4 and 0.8 nm (close to the thickness of two monolayers of water molecules, 0.6 nm), given a particle thickness of 0.94 nm. The cumulative relative frequency of the first peak indicates the relative percentage of intratactoid pore space in the total pores (*f*_intratactoid_). For example, at *f*_Na_ = 1.0 and ρ_d_ = 1000 kg·m^–3^, the inner spacing is about 0.71 nm (Figure 5), and *f*_intratactoid_ falls in the range of 43%–83%, which is consistent with the invasion results (*f*_intratactoid_ = 47%) in Figure 6. The subsequent peaks (labeled 2, 3, 4, and 5 in Figure 7a) correspond to larger nanopores, as illustrated in Figure 7b. For example, peak 2 represents pore widths equivalent to the thickness of one clay layer plus two interplatelet spaces, likely associated with misalignment of platelet edges in individual tactoids.

As noted above based on Figure 2, visual inspection of simulated microstructures reveals little or no sensitivity to counterion type. This observation agrees with the results in Figure 7, where results obtained at *f*_Na_ = 0.2 and 1.0 are broadly consistent. The first peak may be slightly higher in the Ca-rich system, but the statistical significance of this observation was not evaluated here. In contrast, the impact of dry density on nanopore hierarchy is clearly significant: in highly compacted conditions (ρ_d_ = 1800 kg·m^–3^), tertiary, quaternary, and quinary interlayer pores disappear, and the preferred intertactoid distance becomes even more predominant, in agreement with previous studies.^31^ The disappearance of larger pore modes with increasing dry density reflects the collapse of intertactoid pores due to platelet rearrangement and tactoid restructuring. This collapse tends to result in a more uniform pore-size distribution even while compaction, simultaneously, causes a decrease in the number of platelets per tactoid.

### 3.3 Tortuosity

The tortuosity of each system is evaluated using both random walk simulations (eq 2) and the resistor network method (eq 4). The tortuosity obtained from random walk simulations represents the mean tortuosity across the three principal directions, as walkers can freely explore the full 3D microstructure of the clay system. In contrast, the tortuosity derived from the resistor network method is computed separately along each principal direction, and the mean tortuosity (τ_mean_) is then determined using the harmonic mean of these directional values.

As shown in Figure 8, *f*_Na_ has a minor effect on tortuosity compared to the more pronounced influence of porosity. The increase in tortuosity with decreasing porosity is likely caused by the breakup of tactoids due to compaction, which makes large pores more tortuous. For tortuosity predicted by the resistor network method, the tortuosity in the vertical direction (τ_v_) is two to five times larger than the horizontal tortuosity (τ_h_), and this difference becomes greater at low porosities, perhaps because the ratio of intratactoid pores (oriented in the horizontal direction) to intertactoid pores (more variably oriented) increases with compaction, as noted above. Tortuosity values predicted by the resistor network method are consistent with a power-law relation (in agreement with the so-called “Archie’s law” empirical model^83^) with best-fit relationships τ_v_ = 0.82ϕ^–1^,^93^ τ_h_ = 0.99ϕ^–0.11^, and τ_mean_ = 1.04ϕ^–0.37^, as shown by the gray lines in Figure 8.

**Figure 8:**
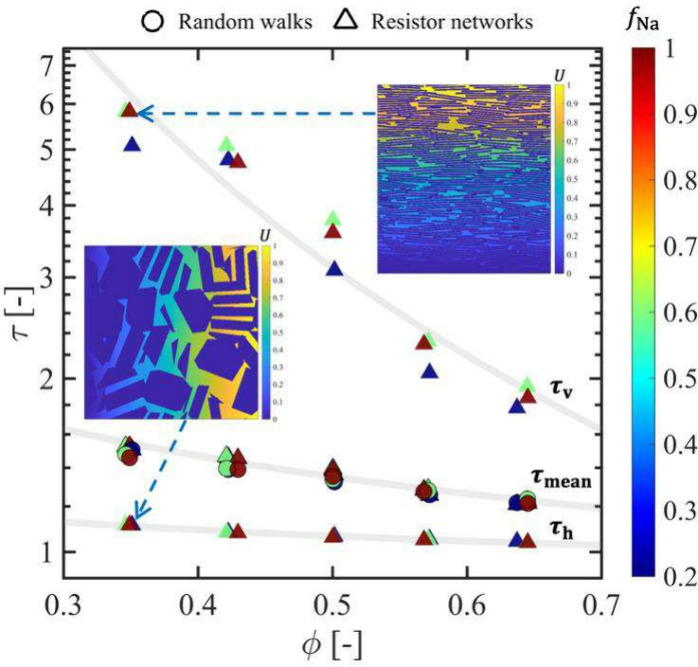
Tortuosity as a function of porosity (ϕ) and Na fraction (*f*_Na_) computed using the random walk (circle markers) and resistor network methods (triangle markers). Upper and lower data series (markers without black edges) correspond to tortuosity in the vertical (τ_v_) and horizontal (τ_h_) directions, respectively, while the intermediate data points (markers with black edges) indicate the average tortuosity (τ_mean_). The gray lines represent the corresponding best-fit power law relations. The inset figures show cross sections of three-dimensional calculations of the equilibrated electrostatic potential distribution for cases when a potential difference (Δ*U* = 1) is applied along the vertical (top) and horizontal directions (bottom). In these examples, ϕ = 0.35, ρ_d_ = 1850 kg·m^–3^, and *f*_Na_ = 1.

The τ_mean_ values predicted by the two methods are consistent, which is expected since both approaches treat the pore space as a purely geometric network. In practice, higher tortuosity would be expected from the diffusion-based method (eq 2) if *D*_a_ were obtained by MD simulations rather than random walk simulations, because water and solutes have lower self-diffusion coefficients within ∼0.5 nm of the clay surfaces.^73,84^ The resistor network method provides a direct and computationally efficient assessment of connectivity, whereas the random walk method excels at capturing complex geometries, including dead-end pores and irregular pathways, though sufficient sampling is required to explore long or narrow conduits. Both methods are sensitive to the grid resolution of the reconstructed systems.

### 3.4 Swelling Pressure

As described in the methodology section, the simulated clay systems are progressively compacted by uniaxial deformation in the *z*-direction. During deformation, the normal stress in the *z*-direction is recorded (i.e., the *P*_*zz*_ component of the internal stress tensor). This normal stress is referred to hereafter as the swelling pressure (*P*_sw_), as it reflects the system’s mechanical response to internal pressure under vertical confinement in drained conditions, in equilibrium with a hypothetical pure liquid water reservoir (i.e., the simulated systems can freely uptake or expel the implicit solvent). The evolution of swelling pressure as a function of dry density is shown in Figure 9 (purple markers), together with a compilation of previous MD simulation predictions of suction (*P*_sc_) (blue markers), experimental suction measurements (green markers), and experimental swelling pressure data (orange markers).^17,44,85–92^ Dashed lines represent regression fits to the blue, purple, and orange data sets based on data for dry densities above 1200 kg·m^–3^.

**Figure 9:**
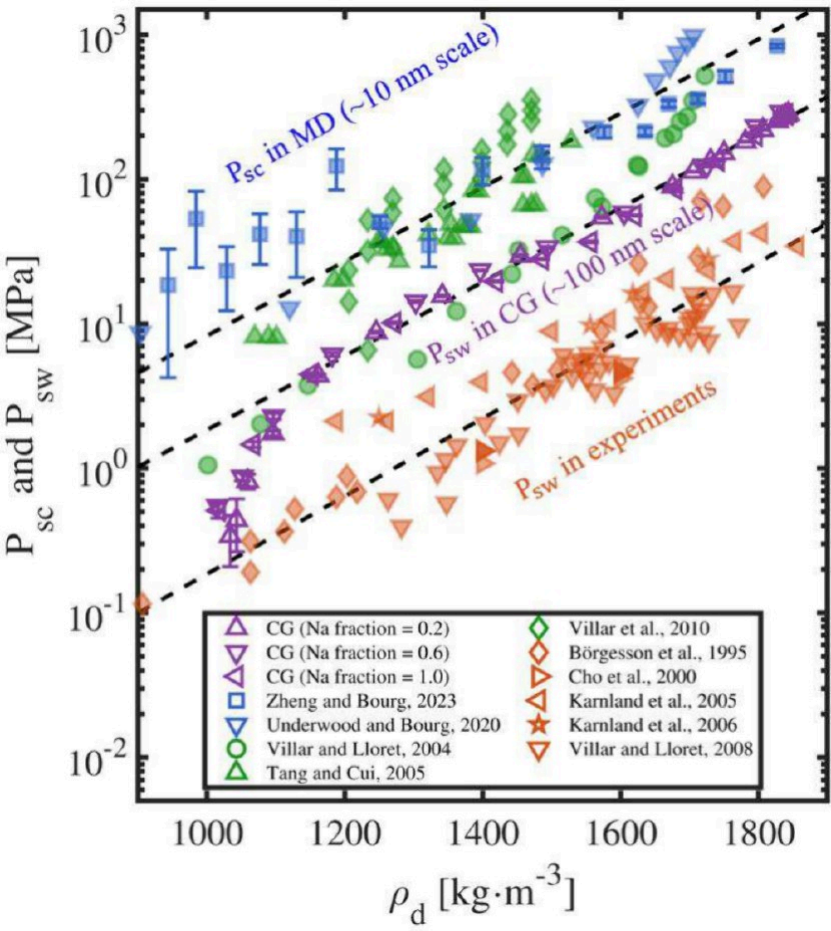
Swelling pressure (*P*_sw_) and suction (*P*_sc_) of smectite and bentonite as a function of dry density (ρ_d_). Blue, purple, orange, and green markers represent previous MD simulation predictions of suction, CG simulation predictions of swelling pressure from this work, experimental swelling pressure results, and experimental suction measurements, respectively.^17,44,85–92^ Dashed lines represent the regression fits for the blue, purple, and orange data sets. Negative suction values from previous MD simulations at low dry densities are omitted. The regression fits were derived using data at dry densities above 1200 kg·m^–3^ to avoid potential bias associated with excluding negative suction values in the MD simulation data sets at lower densities. This figure is modified from Figure 6 in ref 44 to include CG simulation results obtained in this study. Adapted from ref 44. Copyright 2023 American Chemical Society.

The strong increase in both suction and swelling pressure with dry density is expected, as it corresponds to a decrease in basal spacing and a resulting rise in osmotic pressure and electrostatic repulsion due to overlapping electric double layers.^93–95^ In an ideal confined system at equilibrium, *P*_sw_ = *P*_sc_ (i.e., the orange and green experimental markers should overlap).^96,97^ However, as shown in Figure 9, experimental and simulation data reveal that swelling pressure is consistently lower than suction at a given dry density.^98–100^ The regression lines for different data sets are approximately parallel, indicating that the influence of dry density on *P*_sw_ and *P*_sc_ is consistent across different approaches.

The discrepancy between *P*_sw_ and *P*_sc_ likely reflects the difference in characteristic length scales between different simulation and experimental approaches.^101^ A comparison of the regression lines shows that the MD-predicted suction data—obtained from ∼10 nm scale simulations—are consistent with the experimental suction data but 1.6 log units higher than the experimental swelling pressures measured at the centimeter scale (orange markers). In contrast, the CG swelling pressure data obtained in this work (purple markers) are only 0.9 log units higher than the experimental values, in agreement with other recent CG studies.^102^ This shift supports our previous hypothesis that the discrepancy between MD-predicted *P*_sc_ and experimentally measured *P*_sw_ originates from structural heterogeneity that emerges at length scales larger than 10 nm.^22,44^ As the simulated clay system increases in size to include thousands of particles, features such as clay tactoids and macropore formation begin to appear—features that are commonly observed in macroscopic bentonite and smectite samples. This structural heterogeneity enables more realistic predictions of swelling pressure, which are not accessible in small-scale MD simulations involving only a few tens of clay particles. From this trend, we infer that simulation dimensions ∼20 times larger than those achieved in this work (i.e., on the order of 2 μm) may be required to recover experimentally observed macroscale swelling pressures. This would be consistent with the observation that dry clay powders exhibit structure at micrometer scales.^103,104^

## 4 Conclusions

This study demonstrates the strong potential of large-scale coarse-grained (CG) simulations parametrized using all-atom MD simulations for predicting the structural, mechanical, and transport properties of clay-rich geomaterials. By simulating systems containing up to 1,397 montmorillonite particles across a range of dry densities (1031–1843 kg·m^–3^) and counterion compositions (*f*_Na_ = 0.2, 0.6, 1), we systematically investigated a broad set of physicochemical properties, including clay microstructure, pore size distribution, tortuosity, ion diffusivity, and swelling pressure.

This work partially bridges the gap between molecular-scale simulations and macroscale models associated with the complex microstructure of clay particle arrangements on length scales up to micrometers. Earlier efforts were typically constrained by system size (in the case of MD simulations)^17,23,44,50,105^ or required highly simplified interparticle interaction potential models (in the case of CG simulations).^31,102,106^ In contrast, our new CG model, parametrized to match all-atom MD simulation results, captures both nanoscale features and mesoscale heterogeneity, including fluid-filled interlayer nanopores, tactoid formation, anisotropic pore networks, and the emergence of macropores. Notably, the large-scale CG framework partly reconciles discrepancies between suction and swelling pressure by incorporating structural heterogeneity across scales. The reconstructed pore networks yield detailed microstructural features consistent with experimental proxies such as small-angle X-ray scattering (SAXS). Finally, tortuosity estimates from diffusion-based and resistor network methods agree with each other and with microstructural observations of mesopore disappearance and tactoid breakup during compaction.

The ability to reconstruct realistic, three-dimensional pore networks from equilibrated CG configurations offers a robust foundation for predicting macroscale behavior in continuum-scale models. The present results provide quantitative links between particle-scale physics and parameters commonly used in continuum constitutive models for clay-rich geomaterials. For example, the predicted relationships between dry density, basal spacing, tactoid structure, pore size hierarchy, and tortuosity can directly inform anisotropic porosity–permeability and diffusion models.^77,107–109^ This capability opens new avenues for applying molecular simulations to geotechnical, environmental, and energy challenges, including evaluating bentonite barriers for nuclear waste isolation, assessing caprock integrity in CO_2_ storage formations, modeling contaminant transport in landfill liners, and predicting the sensitivity of clay mechanics to aqueous chemistry and particle shape and charge density.

The main limitation to the accuracy of model used here is common to most all-atom or coarse-grained models. Namely, it represents the potential energy of the simulated systems as a sum of pairwise interaction potentials.^110^ Because of this widely used approximation, the coarse-grained interaction potentials used here incorporate multibody effects in an effective manner and their accuracy for other conditions (e.g., other salinities or other clay minerals) will require careful evaluation. Nevertheless, the explicit representation of clay charge sites and counterions included in this model should enable it to capture multibody effects associated with charge screening and the structure of the electrical double layer that play important roles in controlling the properties of clay assemblages.^111–114^ In other words, while the force-field parametrization and results presented in this study are specific to Na/Ca-montmorillonite at zero salinity, the CG framework itself can likely be extended to other counterion types, higher salinities, particle shapes, and smectites with different charge densities. More broadly, it holds promise for the simulation of other layered silicates through appropriate mineral-specific reparameterization, for example, to account for charge originating from tetrahedral substitutions or edge surface protonation or changes in basal surface hydrophilicity due to structural fluorination.^115^

In future work, the present CG framework will be extended to model more realistic clay systems by incorporating particle size polydispersity, spatially heterogeneous charge distributions, and platelet flexibility through relaxation of the rigid-body constraint. A second opportunity for future development consists in extending the model parametrization to include anions, additional cations, organic compounds, tetrahedral charge sites, and ionizable clay edge sites. A third opportunity lies in exploring the feasibility of achieving larger spatial scales by accelerating the computation of long-range Coulomb interactions. Finally, a fourth opportunity lies in implementing a coarse-grained water model to enable representation of unsaturated systems and capillary phenomena.

## Supplementary Material



## Notes

The relationship between scattering intensity and scattering vector magnitude; characteristic microstructural properties of the clay assembly inferred from predicted SAXS results as a function of dry density and Na fraction; mean invaded volume and differential volume contribution as a function of pore width in quasi-mercury intrusion simulations; overall porosity, intertactoid porosity, and intratactoid porosity at different dry densities and Na fractions; and self-diffusion coefficients predicted from random walk simulations (PDF)

## #

X.Z.: School of Civil and Environmental Engineering, Nanyang Technological University, Singapore 639798, Singapore (xiaojin.zheng@ntu.edu.sg)
