# Supplementary material for: Microstructure, Transport, and Mechanics of Compacted Clay Simulated at the 0.1 μm Scale (1400 Smectite Clay Particles) Using a Coarse-Grained Model with Explicit Counterions
Source: J Phys Chem C Nanomater Interfaces. 2026 Mar 3;130(10):3990–4004. doi: 10.1021/acs.jpcc.6c00004 (PMC12990114; doi:10.1021/acs.jpcc.6c00004)
Supplement: Supplementary file 1 [file jp6c00004_si_001.pdf]

Microstructure, Transport, and Mechanics of Compacted Clay Simulated at the 0.1 µm Scale (1400 Smectite Clay Particles) Using a Coarse-Grained Model With Explicit Counterions

Supporting Information

Xiaojin Zheng[1*] and Ian C. Bourg[1,2]

[1] Department of Civil and Environmental Engineering, Princeton University, Princeton, New Jersey 08544

[2] High Meadows Environmental Institute, Princeton University, Princeton, New Jersey 08544

[*] Present address: School of Civil and Environmental Engineering, Nanyang Technological University, Singapore 639798

S1. Relationship between scattering intensity and scattering vector magnitude

Figure 4 in the manuscript shows the shifted scattering intensity (I) as a function of the scattering vector magnitude (q) for systems with f_{Na} = 0.2. The following figure presents the raw data for different f_{Na} conditions.

(a) f_{Na} = 0.2

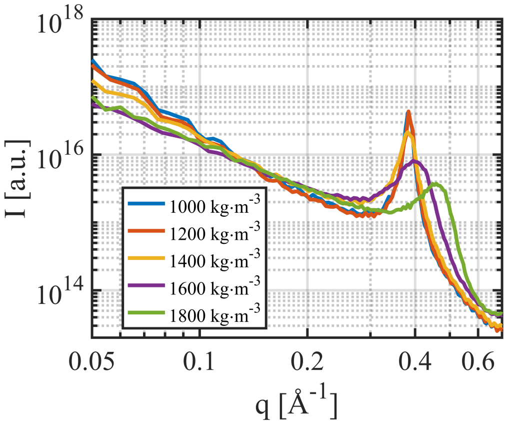

(b) f_{Na} = 0.6

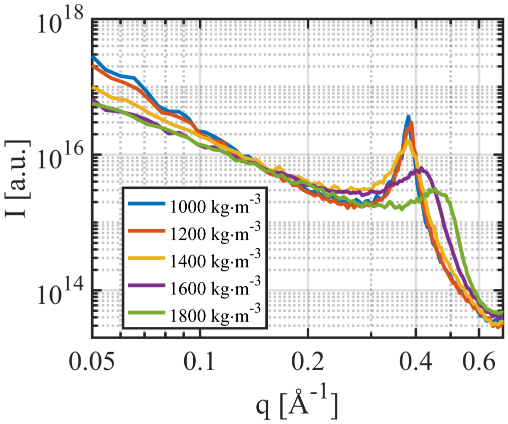

(c) f_{Na} = 1.0

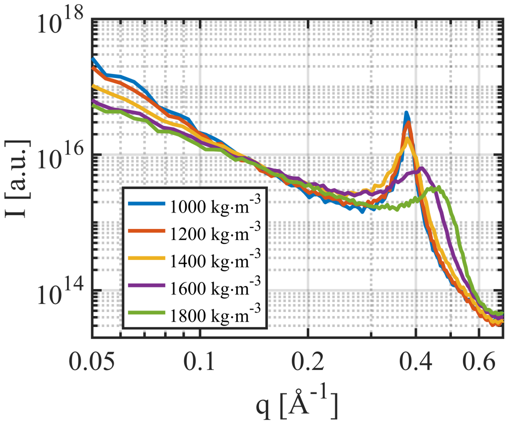

Fig. S1. Scattering intensity (I) as a function of scattering vector magnitude (q) for: (a) systems with f_{Na} = 0.2; (b) systems with f_{Na} = 0.6; and (c) systems with f_{Na} = 1. Different colored lines represent different dry density conditions.

S2. Characteristic microstructural properties

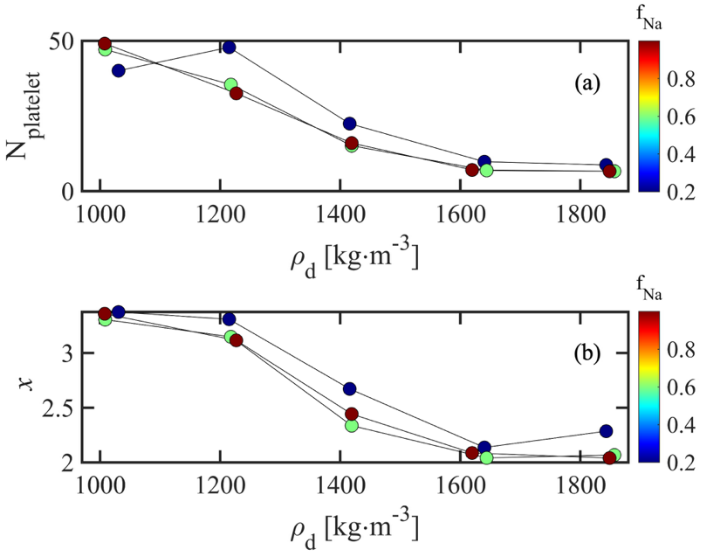

Fig. S2. Characteristic microstructural properties of the clay assembly inferred from predicted SAXS results as a function of dry density (ρ_{d}) and Na fraction (f_{Na}): (a) number of platelets per tactoid (N_{platelet}) and (b) exponent x of the power-law fit in the low-q region.

S3. Quasi-mercury intrusion simulations

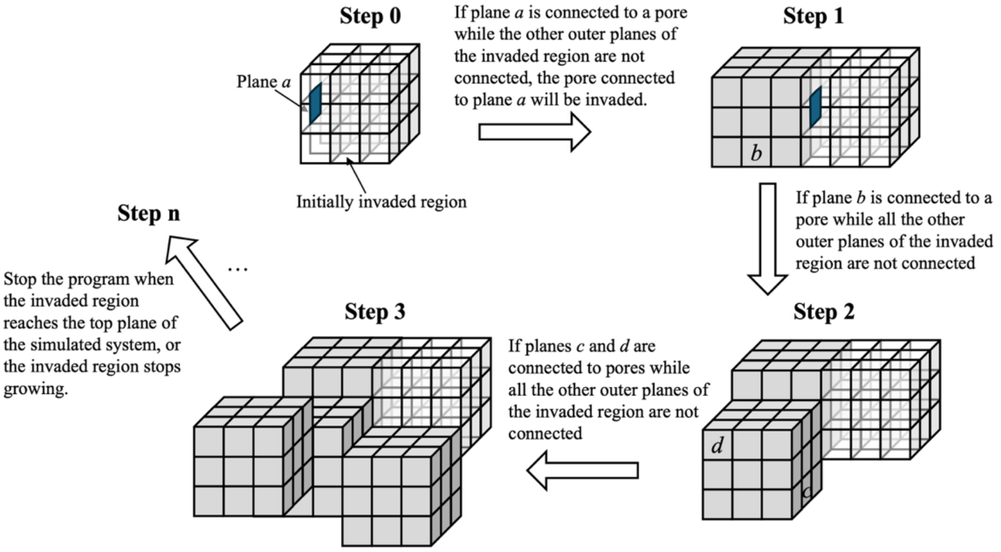

Fig. S3. Schematic diagram showing the workflow of the quasi-mercury intrusion simulation, highlighting the propagation process of the invaded region. In this example workflow, the insertion box is a cube with a side length of 3 cells.

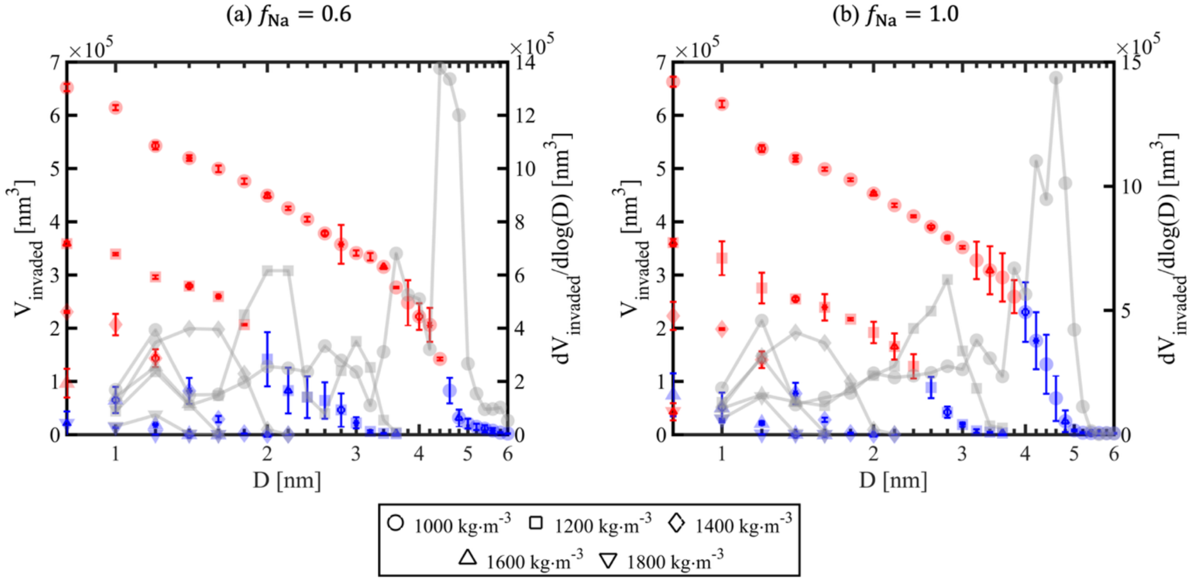

Fig. S4. Mean invaded volume (V_{invaded}) (colored markers) and differential volume contribution ($\mathrm{dV_{invaded}/dlog(D)}$) (grey lines) as a function of pore width (D) (i.e., insertion box size). Each data point represents the average result of 20 insertion attempts, with error bars indicating two standard errors. Red markers indicate cases where the invaded region reaches the top plane—i.e., the invasion process breaks through the simulated system—while blue markers indicate cases where it does not. Panels (a) and (b) correspond to f_{Na} = 0.6 and 1, respectively.

S4. Porosity

Table S1. Overall porosity ($\emptyset$), inter-tactoid porosity ($\emptyset_{\mathrm{inter-tactoid}}$), and intra-tactoid porosity ($\emptyset_{\mathrm{intra-tactoid}}$) at different dry densities and Na fractions. The bounded row represents the average properties across three counterion compositions with ±2 standard errors.

f_{Na}	ρ_{d}	$\emptyset$	$\emptyset_{\mathrm{inter-tactoid}}$	$\emptyset_{\mathrm{intra-tactoid}}$
0.2	1031	0.64	0.30	0.34
0.6	1009	0.64	0.33	0.32
1.0	1008	0.65	0.33	0.31
	1016 ± 15	0.64 ± 0.01	0.32 ± 0.02	0.32 ± 0.02
0.2	1215	0.57	0.21	0.36
0.6	1218	0.57	0.22	0.35
1.0	1227	0.57	0.22	0.35
	1220 ± 7	0.57 ± 0.00	0.22 ± 0.00	0.35 ± 0.01
0.2	1416	0.50	0.14	0.37
0.6	1419	0.50	0.16	0.34
1.0	1419	0.50	0.16	0.34
	1418 ± 2	0.50 ± 0.00	0.15 ± 0.02	0.35 ± 0.02
0.2	1640	0.42	0.05	0.37

0.6	1644	0.42	0.08	0.34
1.0	1620	0.43	0.06	0.37
	1635 ± 15	0.42 ± 0.01	0.06 ± 0.02	0.36 ± 0.02
0.2	1843	0.35	0.03	0.32
0.6	1857	0.35	0.02	0.33
1.0	1849	0.35	0.04	0.31
	1850 ± 8	0.35 ± 0.00	0.03 ± 0.01	0.32 ± 0.01

S5. Self-diffusion coefficients of ions

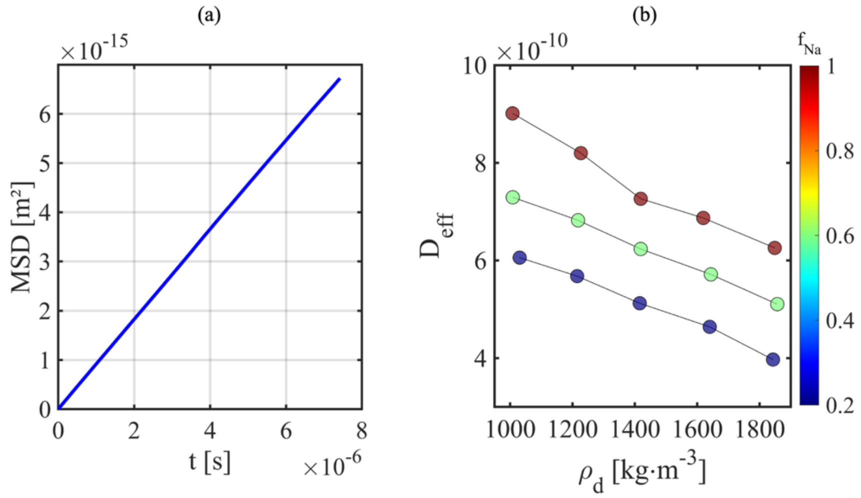

Fig. S5. Self-diffusion coefficients predicted from random walk simulations. (a) Representative plot of mean square displacement (MSD) vs. time at $f_{\mathrm{Na}} = 0.2$ and $\rho_{\mathrm{d}} = 1000\ \mathrm{kg \cdot m^{-3}}$. (b) Self-diffusion coefficients of the Na/Ca ion mixture as a function of dry density (ρ_{d}) and Na fraction (f_{Na}) obtained from random walker simulations.
